# A Clinical Audit to Improve Documentation of Mental Capacity Assessments in Acute Hospital Liaison Teams

**DOI:** 10.1192/bjo.2026.11753

**Published:** 2026-06-30

**Authors:** Oluwafisayo Williams, Bushra Azam

**Affiliations:** Derbyshire Healthcare Foundation Trust, Chesterfield, United Kingdom

## Abstract

**Aims:**

The primary aim was to assess the quality of recording mental capacity assessments by the Chesterfield MHLT to ensure compliance with legal and ethical obligations. The audit aimed for 100% documentation of capacity assessments during all mental health reviews. Additionally, the project targeted a minimum Grade 2 standard (moderate detail) in documenting the four functional stages of capacity for all patients, ensuring that narrative and context regarding a patient’s capacity component were thoroughly recorded.

**Methods:**

A retrospective review of 30 randomized electronic patient records (electronic system) was conducted for the Chesterfield MHLT for cases identified between January and February 2025. Data was analysed to determine the presence of broad capacity assessments and, crucially, to evaluate the quality of documentation regarding the four functional stages: Understanding, Retaining, Weighing-up, and Communicating information. A standardized grading scale (Grade 0-3) was utilized to measure the depth of narrative and the presence of direct patient quotes in the documentation

**Results:**

While a broad assessment of capacity was recorded in 90% of cases, the documentation quality regarding specific domains was inadequate. Only 37% of cases explicitly documented the specific decision being assessed, such as consent to medication or admission, representing a critical gap in compliance. Furthermore, the detailed assessment of the functional stages frequently failed to meet the required standard. For the functional domains of “Understanding” and “Communicating,” 50% of cases showed no evidence (Grade 0) of documentation. Similarly, 40% of cases showed no evidence of documentation for “Weighing-up” information, highlighting a significant discrepancy between broad capacity recording and the required detailed documentation of the assessment process

**Conclusion:**

This audit highlights that while capacity is often broadly recorded, the specific, detailed assessment of how patients understand, retain, weigh up information, and communicate their decisions is frequently absent or insufficient. To enhance compliance, we recommend integrating mandatory MCA forms into the core assessment template on electronic and enforcing explicit, decision-specific documentation. A re-audit is planned in six months to measure the impact of these changes on documentation quality and patient autonomy.

